# Critical Role of Extracellular Vesicles in Diffuse Large B-Cell Lymphoma; Pathogenesis, Potential Biomarkers, and Targeted Therapy—A Narrative Review

**DOI:** 10.3390/biomedicines12122822

**Published:** 2024-12-12

**Authors:** Teerachat Punnachet, Siriporn C. Chattipakorn, Nipon Chattipakorn, Sirinart Kumfu

**Affiliations:** 1 Division of Hematology, Department of Internal Medicine, Faculty of Medicine, Chiang Mai University, Chiang Mai 50200, Thailand; teerachat_p@outlook.com; 2 Cardiac Electrophysiology Research and Training Center, Faculty of Medicine, Chiang Mai University, Chiang Mai 50200, Thailand; 3 Center of Excellence in Cardiac Electrophysiology Research, Chiang Mai University, Chiang Mai 50200, Thailand; 4 Cardiac Electrophysiology Unit, Department of Physiology, Faculty of Medicine, Chiang Mai University, Chiang Mai 50200, Thailand

**Keywords:** diffuse large B-cell lymphoma, extracellular vesicles, miRNA, tumor microenvironment, biomarker

## Abstract

Diffuse large B-cell lymphoma (DLBCL) is the most common subtype of non-Hodgkin’s lymphoma, characterized by its aggressive nature and heterogeneity. Despite significant advances in understanding DLBCL pathogenesis, there is still a need to elucidate the intricate mechanisms involved in disease progression and identify novel therapeutic targets. Extracellular vesicles (EVs), including exosomes and microvesicles, have emerged as crucial mediators of intercellular communication in various physiological and pathological processes, including cancer. In recent years, evidence has suggested that EVs play a vital role in DLBCL biology by facilitating the exchange of genetic material, especially miRNAs, and proteins and lipids between tumor cells, immune cells, and the tumor microenvironment. We summarize and discuss the biological functions of EVs in DLBCL and their effects on the tumor microenvironment, highlighting their influence on DLBCL pathobiology, immune evasion, angiogenesis, and drug resistance. We also investigated EVs’ diagnostic and prognostic potential as circulating biomarkers in DLBCL, emphasizing their utility in the non-invasive monitoring of the disease status and treatment response. Understanding the complex interplay between EVs and DLBCL may open up new avenues for personalized medicine, improve patient stratification, and facilitate the development of innovative therapeutic interventions in this devastating hematological malignancy.

## 1. Introduction

Lymphoma comprises a heterogeneous group of cancers that arise from the clonal proliferation of lymphocytes. Lymphoma is categorized into two broad groups: Hodgkin’s lymphoma (HL) and non-Hodgkin’s lymphoma (NHL). Within NHL, diffuse large B-cell lymphoma (DLBCL) is the most common subtype, accounting for approximately 30% of cases [1]. R-CHOP (rituximab, doxorubicin, cyclophosphamide, vincristine, and prednisone) immunochemotherapy has been considered the standard first-line care for DLBCL. However, only two-thirds of patients can be cured [2,3,4] and the rest are either refractory or experiencing a relapse, highlighting the need for a better understanding of the biology of the disease and also the identification of novel therapeutic targets. Despite advances in our understanding of DLBCL, the underlying mechanisms driving its pathogenesis and treatment resistance are complex and not fully understood.

In the past decades, the study of extracellular vesicles (EVs) has been rapidly expanded and continuously updated. Guidelines for studies on EVs by the International Society for Extracellular Vesicles have been published to guide researchers with the aim of reducing heterogeneity, raising standards, and improving the comparability of the studies [5,6]. EVs are lipid-bound, nano-sized vesicles that are secreted by almost all cell types into the extracellular space [7,8]. EVs can be found in various biofluids, most significantly in the blood. EVs are known to carry various bioactive molecules, including lipids, proteins, nucleic acids (DNA, RNA), and other signaling molecules, which are selectively packaged by the parental cells [8]. By delivering their cargoes, EVs play an important role in cell-to-cell communication and thereby influencing diverse effects on the target cells, from stimulating signaling pathways to providing trophic support.

There is growing evidence that EVs play a central role in cancer development and progression [9,10]. However, the role of EVs in DLBCL remains largely unknown and hence necessitates further exploration. Understanding EVs may lead to promising new therapeutic approaches. The purpose of this review is an attempt to summarize our current understanding of the potential role of EVs in DLBCL, their role in biological functions, their association with tumor-associated macrophages, and their clinicopathologic features, diagnostic markers, prognostic prediction value, and treatment efficacy. In vitro, in vivo, and clinical studies have been included in this review.

## 2. Searching Methodology and Selection Criteria

A comprehensive search of the literature was performed using PubMed. The literature search included only articles written in English. An article was rejected if it was a letter or case report. The search used the following keywords: exosomes; extracellular vesicles; diffuse large B-cell lymphoma; and DLBCL either in the title, abstracts, or text. The relevance of the subject and eligibility of all publications detected were further evaluated, and data were then extracted from relevant papers to be included in this review.

## 3. Biological Functions of DLBCL-Cell-Derived EVs

EVs are secreted by tumor cells and are commonly known as tumor-derived EVs. They exert their biological functions by delivering their cargoes to target cells, the mediators of cell-to-cell communication [9]. DLBCL-derived EVs carry biomolecules that can promote tumor progression and survival by increasing cell proliferation, invasion, and chemoresistance, and also by decreasing cell apoptosis [11,12,13,14,15,16,17]. Wnt signaling is a critical cellular signaling pathway that plays a fundamental role in the development and maintenance of homeostasis within tissues. It has been postulated that the deregulation of Wnt signaling plays a crucial role in lymphoma pathogenesis and progression [18,19]. Koch R et al. demonstrated that DLBCL has a self-organized infrastructure comprising side population (SP) and non-SP cells and that this transition between clonogenic states is regulated by EV-mediated Wnt signaling [20]. There is also increasing evidence to support the hypothesis that EVs can modulate chemoresistance in various cancers [21]. In the case of DLBCL, EVs derived from DLBCL cells have been shown to induce chemoresistant properties in DLBCL cells through various mechanisms. MicroRNAs (miRNAs) carried by EVs participate in post-transcriptional regulation. Studies have shown that both the increased and decreased expression of miRNAs can impact tumor progression, depending on the effects of the target genes. Rituximab is an anti-CD20 chimeric monoclonal antibody that binds the CD20 antigen on DLBCL cells and has been approved for the treatment of DLBCL. To escape and reduce the efficacy of rituximab, EVs reduce CD20 expression in the lymphoma cells through the regulation of gene expression via exosomal shuttling microRNA-125b-5p [11]. Other mechanisms of EV-mediated resistance to rituximab include the absorption of the rituximab itself and consumption of the complement [17,22]. Interestingly, DLBCL cells can also release cytostatic chemotherapy drugs such as doxorubicin and pixantrone by packaging them into exosomes, which are then exported out of the cells, contributing to decreased drug retention [16]. Tandem mass tag labeling to enable the quantitative proteomics analysis of proteins extracted from serum-derived exosomes from DLBCL patients shows the upregulation of carbonic anhydrase 1 (CA1) [12]. This finding is consistent with the increase in the CA1 protein determined by the Western blot analysis of exosomes from chemoresistant DLBCL cells. The study in question also demonstrated that DLBCL exosomal CA1 has been shown to induce chemoresistance via the promotion of the NF-kB and STAT3 signaling pathways. The constitutive activation of nuclear factor κB (NF-κB) is one of the key signaling pathways involved in DLBCL growth and survival, particularly in the case of the activated B-cell-like (ABC) subtype [23,24]. Moreover, DLBCL-derived EVs also play a role in mediating crosstalk with the tumor microenvironment and might promote tumor progression by conditioning macrophages [14]. DLBCL-derived EVs have been found to contain NSE (neuron-specific enolase), a protein that is primarily associated with neurons in the central nervous system. However, recent studies have shown that NSE can also be found in other tissues, including cancer cells. In the context of DLBCL, NSE-containing EVs may play a role in promoting the survival of lymphoma cells and their growth by modulating interactions with macrophages [15]. The details of DLBCL-derived EVs and their association with macrophages will be discussed in the following section.

Intriguingly, EVs have been shown to have both positive and negative effects. Lobastova L et al. showed that CD30-positive EVs can mediate cell damage to CD30-negative cells when combined with brentuximab vedotin, an anti-CD30 antibody-drug conjugate [25]. This finding suggests that EVs can potentially be utilized in a beneficial manner, such as in enhancing the efficacy of targeted therapies like antibody-drug conjugates.

**Figure 1:**
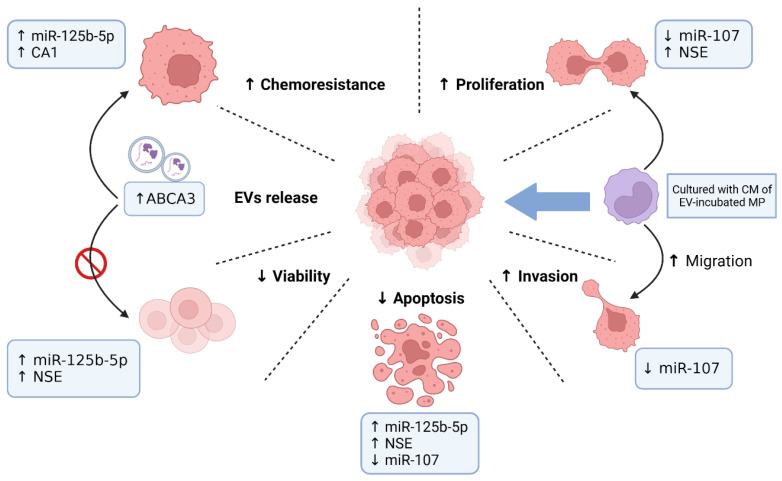
Biological functions of DLBCL-cell-derived EVs. ABCA3, ATP-transporter A3; CA1, carbonic anhydrase 1; CM, conditional medium; EVs, extracellular vesicles; NSE, neuron-specific enolase. ↑: increase or higher; ↓: decrease or lower; 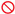: inhibit.

Indeed, DLBCL-derived EVs have been shown to have a dual role in promoting tumor survival while also potentially enhancing the effect of monoclonal antibody therapy. Therefore, targeting DLBCL-derived EVs could be a promising strategy for the treatment of DLBCL. By inhibiting the release or uptake of these EVs, or by neutralizing their cargo, it may be possible to disrupt the pro-tumor effects of DLBCL-derived EVs and enhance the efficacy of other anti-cancer treatments, including monoclonal antibodies. Reports on these in vitro findings are summarized in Table 1 and Figure 1.

## 4. Effects of DLBCL-Cell-Derived EVs on Macrophages

The tumor microenvironment is a complex milieu in which cancer cells interact with various cells and cellular components, including immune cells, stromal fibroblasts, endothelial cells, and the extracellular matrix [26]. In the case of DLBCL, the disease is not solely driven by autonomous cell growth but also relies on signals for survival and proliferation from the tumor microenvironment [27,28,29]. Tumor-associated macrophages (TAMs) play a crucial role in the tumor microenvironment and are associated with treatment outcomes and prognostic implications in lymphomas including DLBCL [30,31,32,33,34]. Macrophages can be categorized into two functionally distinctive polarization states, M1 and M2 macrophages, depending on the microenvironmental stimuli. M1 macrophages exhibit an anti-tumor effect and are characterized by an increased ability to secrete pro-inflammatory cytokines such as IL-1β, IL-12, IL-18, and TNF. Phenotypically, M1 macrophages express high levels of major histocompatibility complex class II (MHC-II), CD68, CD80, and CD86 co-stimulatory molecules. On the other hand, common markers for M2 macrophages are CD206, CD204, and CD163. M2 macrophages secrete various cytokines such as TGF-β and IL-10, which can promote tumor cell proliferation and inhibit the anti-tumor activity of T cells and NK cells [35,36]. Molecular transfer via EVs among tumor cells and TAMs is one possible mechanism that may connect a tumor and educate macrophages, consequently promoting tumor progression and drug resistance. DLBCL-cell-derived EVs can regulate macrophages into the M2 phenotype by increasing the expression of the protein PGC-1β, which in turn promotes tumor progression [14]. NSE expression is increased and associated with poor prognosis in DLBCL patients, particularly in the case of non-germinal center B-cell type [37,38,39]. EVs containing NSE can promote M2 polarization by disrupting the NF-kB pathway and enhancing the migratory capability of macrophages, thereby promoting tumor proliferation [15]. The miRNAs contained within EVs can be delivered to macrophages and regulate the expression of various genes. One such miRNA is miR-7e-5p, which can be transported from DLBCL cells to M1 macrophages. One study has found that miR-7e-5p was downregulated in high-grade follicular lymphoma (FL) and DLBCL when compared to indolent FL. The decreased expression of miR-7e-5p in tumor cells resulted in a reduced transfer of miR-7e-5p through EVs, leading to the upregulation of FASL and apoptotic caspase signaling in activated M1 macrophages [40].

To conclude, DLBCL-derived EVs carry various cargoes, e.g., NSE and miRNAs, that can modulate the interactions between lymphoma cells and macrophages in the tumor microenvironment. These EVs can regulate macrophage polarization into the M2 phenotype, which promotes tumor progression and drug resistance and can also deliver miRNAs to M1 macrophages to activate apoptotic caspase signaling. As our understanding of the relationship between TAMs and DLBCL deepens, TAMs could have the potential to emerge as a potential novel therapeutic target. Therapeutic strategies that target or reprogram macrophages through EVs hold promise as a potential therapeutic approach to improve treatment outcomes [41]. By leveraging the role of EVs in mediating communication between tumor cells and macrophages, it may be possible to develop innovative therapies that disrupt this crosstalk and reprogram macrophages to exert anti-tumor effects, thereby improving the management of DLBCL and potentially other cancers as well. The effects of DLBCL-cell-derived EVs on macrophages are summarized in Table 2.

**Figure 2:**
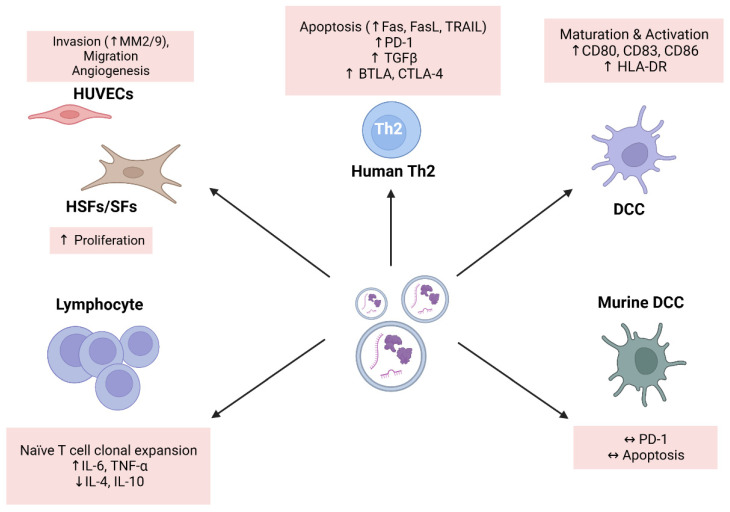
Communication between EVs and recipient cells. Abbreviations: DCCs, dendritic cells; HSFs, human skin fibroblasts; HUVECs, human umbilical vein endothelial cells; MM, matrix metalloproteinases; SFs, primary gastric-related fibroblasts. ↑: increase or higher; ↓: decrease or lower; ↔: no change or equal.

EVs have been shown to mediate both pathogenic and protective effects in DLBCL. Chen et al. demonstrated the dual role of DLBCL-derived EVs [42]. On one hand, EVs promote tumor progression by enhancing the invasion, migration, and angiogenesis of human umbilical vein endothelial cells, stimulating the proliferation of BM stromal cells and fibroblast cells, and inducing the apoptosis of Th2 cells. On the other hand, EVs can mediate anti-tumor effects through dendritic cells stimulated by EVs, as demonstrated by their ability to stimulate the clonal expansion of T cells, increase the secretion of pro-inflammatory cytokines, and decrease the production of immunosuppressive factors. These findings highlight the potential for EVs to improve future novel immunotherapies. The relevant study is summarized in Figure 2.

## 5. Role of DLBCL-Cell-Derived Evs: Reports from In Vivo Studies

The majority of the in vivo studies were carried out using subcutaneously implanted DLBCL cells in mice. A study reported that the coinjection of DLBCL cells with DLBCL-derived Evs in vivo resulted in enhanced tumor growth [42]. This study also provided additional proof regarding the concept of dendritic-cell-based vaccines. Dendritic cells activated by Evs (DCC^EV^) were injected into the mice before being challenged with DLBCL cells. The lymphocytes subsequently obtained demonstrated anti-lymphoma activity. In vivo studies have also confirmed the role of CA1, miR-125b-5p, miR-107, and NSE in tumor progression and chemoresistance as demonstrated by increased tumor sizes, migration, angiogenesis, and reduced sensitivity to chemotherapy [11,12,13,15].

Targeting EV formation and release from DLBCL may have potential clinical therapeutic implications [17]. The majority of anti-cancer drugs function as toxins that target nuclear DNA to cause damage. A combination of chemo-immunotherapy with rituximab remains the mainstay backbone of treatment. Doxorubicin is particularly effective due to its cytostatic efficacy by inhibiting topoisomerase II in the nuclei of tumor cells. Studies have discovered that lymphoma cells can extrude doxorubicin and pixantrone, a topoisomerase II inhibitor, packaged in exosomes [16]. The silencing of ABC transporter A3 can suppress the release of Evs, and this pathway is targetable by a cyclooxygenase inhibitor (indomethacin). In a chorioallantoic membrane assay, pretreatment with indomethacin led to increasing drug retention in lymphoma cells, resulting in increased drug efficacy.

**Figure 3:**
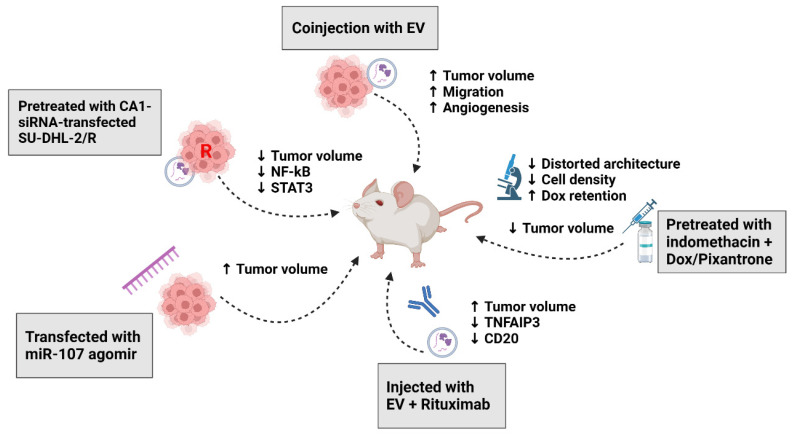
Role of DLBCL-cell-derived Evs: report from in vivo studies. Abbreviations: CA1, carbonic anhydrase 1; Dox, doxorubicin; STAT3, signal transducer and activator of transcription 3; TNFAIP3, tumor-necrosis-factor-alpha-induced protein 3. ↑: increase or higher; ↓: decrease or lower.

The in vivo studies confirmed the pathogenic effects of DLBCL-derived Evs, as seen in the promotion of tumor growth and invasiveness, as well as the role of specific EV cargoes, such as CA1, miR-125b-5p, miR-107, and NSE, in tumor progression and chemoresistance. In addition, the studies also provided evidence for the potential therapeutic use of Evs, particularly through dendritic-cell-based vaccines. The ability to suppress the release of Evs through targeting the ABC transporter A3 pathway with indomethacin also highlights the potential implications for clinical therapy. These in vivo studies are summarized in Table 3 and Figure 3.

## 6. Association Between Circulating Evs and Clinicopathological Features

Several studies have investigated the correlations between the molecular profiles of EVs and clinicopathological characteristics in DLBCL patients, but only a few studies have shown significant correlations (Table 4) [12,13,43,44,45,46]. The programmed cell death ligand (PD-L1) is an immune checkpoint molecule that might play a pivotal role in lymphoma immune escape and is expressed not only on the surfaces of tumor cells but also on exosomes [47,48]. Higher levels of CD9-CD63 and PD-L1-CD63 signals have been associated with the presence of ≥2 extranodal sites, indicating a potential role of EVs in disease dissemination [43]. In addition, one study demonstrated that the lower expression of exosomal miR-107 correlated with poor prognosis, as indicated by an increased ECOG score, stage, and IPI, LDH, and beta-2 microglobulin levels [13]. Another study reported that the higher expression of exosomal CA1 was associated with an increased IPI, suggesting a possible link between CA1 and disease aggressiveness [12]. These findings are consistent with those from both in vitro and in vivo studies. However, no significant correlations were observed between the expression of miR-146a-5p, miR-155, Let-7g, Let-7i, miR-99a-5p, and miR-125b-5p and clinicopathological features in DLBCL patients.

In conclusion, PD-L1 has been detected in both tumor cells and exosomes, with higher levels of CD9-CD63 and PD-L1-CD63 signaling being associated with the presence of extranodal sites. Additionally, the lower expression of exosomal miR-107 has been linked to poor prognosis while the higher expression of exosomal CA1 has been associated with increased disease aggressiveness. Those molecules could be interesting targets for improving treatment in DLBCL patients.

## 7. EVs as a Potential Diagnostic Marker

Over the past decade, the use of liquid biopsy has gained significant attention as a simple and non-invasive approach for cancer diagnosis and treatment monitoring. This technique allows the detection of tumor-derived entities such as circulating tumor DNA, and circulating tumor cells through biofluids, in particular blood. Liquid biopsy has already shown promising clinical applications in various cancers including non-small cell lung cancer, ovarian cancer, breast cancer, and prostate cancer [49]. The properties of EVs provide the benefit of protecting their cargo against degradation by various enzymes, thus making them more attractive for liquid biopsy due to their stability in different conditions [50]. A recent study has demonstrated that plasma-derived EVs from DLBCL patients contain distinct protein signatures that differentiate them from healthy individuals. Using mass spectrometry, the study analyzed the proteomes of plasma-derived EVs, from DLBCL patients and healthy donors, identifying significant differences in the EV proteome. These differences included unique proteins associated with DLBCL, which demonstrated potential as biomarkers for diagnosis [51]. Other studies have revealed that EVs can be traced back to their cells of origin in different B-cell lymphoma cell lines, and mutational profiles of EVs-derived RNAs reflect the genomic landscape in the cell of origin [22,52]. This suggests that DLBCL-derived EVs could potentially serve as diagnostic markers, providing valuable clinicopathological features and therapeutic monitoring. Several studies have investigated the potential role of EVs in DLBCL diagnosis [13,43,44,53,54]. Two studies have reported discriminative performance using EV-miRNAs (miR-107, miR-375-3p, miR-485-3p, and miR-451a), with area under the curve (AUC) values ranging from 0.71 to 0.85 [13,53]. One report using CD9-CD63 EVs (universal EVs) and PD-L1-CD63 EVs (PD-L1+EVs) demonstrated AUC values of 0.99 and 0.87, respectively [43]. These findings suggested that these EVs might be suitable for use as diagnostic indices for DLBCL patients. However, not all miRNAs have shown diagnostic performance in DLBCL. Two studies did not find significant diagnostic values for miR-146a-5p and miR-155 [44,54]. These findings highlight the need for the careful evaluation of specific EV-associated markers.

Overall, EV-miRNAs have shown promising results as potential diagnostic markers for DLBCL, with several studies reporting discriminative performance using specific miRNAs. However, not all miRNAs have shown significant diagnostic value in DLBCL. A summary of these reports is shown in Table 5.

## 8. The Prognostic Prediction Value and Treatment Efficacy of EVs

Emerging evidence suggests that EVs and their cargoes hold promise as novel prognostic markers and tools for treatment monitoring in DLBCL. However, it is important to note that not only tumor cells but also normal cells release EVs, making it crucial to carefully evaluate specific markers and differentiate them from those from physiological conditions. Among the molecules studied inside EVs, miRNAs have been extensively investigated. Studies have demonstrated the dynamic role of EVs in assessing treatment efficacy. Changes in the EV cargo during therapy, such as shifts in miRNA levels or protein composition, have been linked to patient responses. For instance, specific alterations in exosomal protein profiles during chemotherapy have been associated with treatment outcomes, suggesting that monitoring EV cargo could provide real-time insights into therapeutic efficacy. Such findings underscore the potential of EVs not only as biomarkers for disease monitoring but also as indicators of therapeutic response and resistance. The markers of clinical and histopathological parameters such as the IPI score and LDH level have a prognostic impact on the disease. The treatment response was assessed via computed tomography (CT)/magnetic resonance imaging (MRI) or positron emission tomography–computed tomography (PET/CT). The response to chemotherapy was evaluated according to response criteria for lymphoma, including complete response (CR), partial response (PR), stable disease (SD), and progressive disease (PD). As summarized in Table 6, studies have demonstrated that high levels of expression of miR-99a-5p, miR-125b-5p, and miR-155, as well as the low expression of miR-107, are correlated with poor prognosis in DLBCL patients [13,45,46]. The expression of miR-99a-5p and miR-125b-5p was significantly higher in chemoresistant patients (stable or progressive disease) compared to chemosensitive patients (complete or partial response) [46]. ROC curve analyses showed that the AUC values of miR-99a-5p and miR-125b-5p were 0.744 and 0.780, respectively. Combining exosomal miR-99a-5p or miR-125b-5p with the international prognostic index (IPI) score improved accuracy further. Another study reported that miR-155 was significantly increased in relapsed and refractory patients compared to patients who responded to chemotherapy or were receiving chemotherapy [45]. The Let-7 family of miRNAs has been implicated in carcinogenesis and drug resistance in hematologic malignancies including DLBCL [55]. In a study by Zare et al., exosomal Let-7g was significantly decreased in patients who were still ongoing or responsive to treatment while no difference was found with Let-7i [45]. Similar to miR-99a-5p and miR-125b-5p, a higher expression of CA1 was observed in chemoresistant patients (AUC 0.812) and combining CA1 expression with IPI score slightly improved accuracy (AUC 0.821) [12]. The high expression of exosomal CA1 was also associated with inferior progression-free survival (PFS). Furthermore, a study utilizing SiMoa to detect CD9-CD63 and PD-L1-CD63 signals on EVs demonstrated that higher signal levels correlated with worse PFS and treatment response as defined by complete response [43].

In conclusion, the decreased expression of miR-107 and increased expression of miR-99a-5p, miR-125b-5p, miR-155, CA1, CD9-CD63, and PD-L1-CD63 have been shown to correlate with poor prognosis in DLBCL patients. Changes in the expression levels of Let7g and miR-451a may serve as predictors of treatment response. These findings suggest that EVs have the potential to serve as indicators for therapeutic response and prognosis markers for DLBCL patients. However, further research is needed to validate and standardize the use of EVs in DLBCL diagnosis and treatment monitoring and to identify additional markers for improved accuracy and clinical utility.

## 9. Therapeutic Implications

The potential for EVs to serve as therapeutic targets in DLBCL is increasingly supported by emerging evidence. Their role in mediating tumor progression, immune modulation, and drug resistance provides multiple avenues for intervention.

### 9.1. Targeting EV Biogenesis and Release

One promising approach is the inhibition of EV formation and release. Studies have demonstrated that ATP-binding cassette transporter A3 (ABCA3) mediates EV secretion, which can facilitate drug resistance by extruding chemotherapy agents such as doxorubicin and pixantrone [16]. Targeting this pathway with indomethacin, a cyclooxygenase inhibitor, has been shown to reduce EV release, increase drug retention within tumor cells, and enhance therapeutic efficacy in preclinical models. These findings suggest that modulating EV production could improve chemotherapy outcomes in DLBCL.

### 9.2. Blocking EV Uptake

Another therapeutic strategy involves inhibiting the uptake of tumor-derived EVs by recipient cells in the tumor microenvironment. Blocking EV internalization could disrupt the transfer of oncogenic miRNAs and proteins that promote tumor growth, immune evasion, and chemoresistance. Although specific inhibitors of EV uptake are still under investigation, this approach holds promise for preventing the spread of pro-tumorigenic signals.

### 9.3. Neutralizing EV Cargo

Neutralizing specific EV cargo represents a direct therapeutic approach. For instance, the transfer of miR-125b-5p via EVs has been implicated in reducing CD20 expression, thereby contributing to resistance against rituximab therapy. Therapeutic strategies aimed at targeting miR-125b-5p may help restore sensitivity to rituximab [11]. Similarly, exosomal carbonic anhydrase 1 (CA1) has been shown to drive chemoresistance through the activation of NF-κB and STAT3 signaling pathways [12]. Suppressing CA1 activity could potentially reverse resistance mechanisms and enhance treatment outcomes, highlighting the clinical potential of neutralizing specific EV cargoes in improving therapeutic efficacy.

### 9.4. Enhancing Immune Response

EVs also offer opportunities to enhance the immune response against DLBCL. Dendritic-cell-based vaccines pulsed with tumor-derived EVs (DCCEVs) have shown potential in preclinical studies. These vaccines activate cytotoxic T cells and reduce regulatory T cell populations, resulting in anti-tumor activity [42]. Additionally, EVs carrying CD30 have been utilized to extend the efficacy of brentuximab vedotin, an antibody–drug conjugate, enabling the targeting of CD30-negative lymphoma cells [25].

### 9.5. Potential in Combination Therapies

In addition to R-CHOP, which is the first-line treatment in DLBCL, cancer immunotherapy is an exciting novel therapy to treat these patients that include bispecific antibodies (BsAbs) and chimeric antigen receptor T cell therapy (CAR-T) [56]. They are created by re-engineering patient T cells to produce chimeric antigen receptors, or CARs, which are proteins on their surfaces. Certain proteins, or antigens, on the surfaces of cancer cells are recognized and bound by CARs. Patients who progress on or are unable to receive CAR-T now have active options for BsAbs. In order to extend the lives of patients with DLBCL, patients may be able to receive multiple BsAbs or CAR-T during their cancer treatment journeys, each with a different target. Combining EV-targeting strategies with existing therapies, such as R-CHOP or novel immunotherapies like BsAbs and CAR-T cells, could provide synergistic effects. By reducing the pro-tumor effects of EVs, these combination therapies may improve response rates and overcome resistance mechanisms.

## 10. Challenges in Translating EV-Based Approaches to Clinical Settings

Despite the promising potential of EVs as diagnostic and therapeutic tools in DLBCL, several challenges hinder their clinical application. The clinical application of EVs in DLBCL faces several challenges. The lack of standardization in EV isolation and characterization methods complicates the comparability of results across studies and diminishes their clinical reproducibility [57,58]. Identifying reliable biomarkers is complicated by the biological heterogeneity of EVs, and their therapeutic use requires a thorough evaluation of safety, biodistribution, and potential off-target effects. Moreover, it is important to establish proper handling, storage, and transportation conditions for EV-based therapeutics to maintain their structure and functionality. Addressing these challenges will pave the way for integrating EVs into the clinical management of DLBCL and other malignancies.

## 11. Conclusions and Future Perspectives

**Figure 4:**
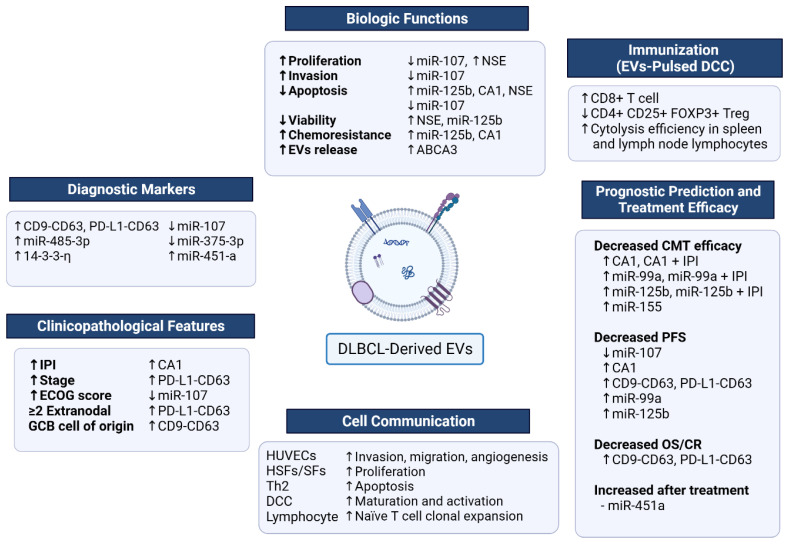
Summary of the potential roles of EVs in DLBCL including their role in biological functions, immunization, cell communication, clinicopathologic features, diagnostic markers, prognostic prediction value, and treatment efficacy from currently available reports. Abbreviations: ABCA3, ATP-transporter A3; CA1, carbonic anhydrase 1; CMT, chemotherapy; CR, complete remission; DCC, dendritic cells; ECOG, Eastern Cooperative Oncology Group; EVs, extracellular vesicles; GCB, germinal center B-cell like; HSFs, human skin fibroblasts; HUVECs, human umbilical vein endothelial cells; IPI, International Prognostic Index; NSE, neuron-specific enolase; OS, overall survival; PFS, progression-free survival; SFs, primary gastric-related fibroblasts; ↑: increase or higher; ↓: decrease or lower.

Emerging evidence suggests that EVs play a crucial role in DLBCL pathogenesis by promoting tumor growth, migration, angiogenesis, immune evasion, and drug resistance. Both DLBCL-derived EVs and EVs from the tumor microenvironment contribute to the complexity of the disease and its microenvironment. These EVs can promote tumor cell proliferation via the NF-kB-STAT3 signaling pathway and regulate macrophage polarization into the M2 phenotype, which promotes tumor progression and drug resistance. Moreover, EVs can increase tumor invasion and migration via the increase in MMP2/9 and increased angiogenesis in HUVECs. The EV cargo comprising components such as miRNA and proteins related to pathogenesis in DLBCL is shown in Figure 4. EVs do have the potential to serve as diagnostic markers and prognostic predictors in DLBCL, with studies showing that specific EV cargoes could be used for disease monitoring and predicting treatment response. Moreover, EVs have also shown promise as potential therapeutic targets in DLBCL, but further research is needed to better understand the mechanisms underlying EV-mediated effects in DLBCL. A summary of the potential roles of EVs is shown in Figure 4. Specifically, more studies are required to elucidate the specific cargo molecules and signaling pathways involved in the effects of DLBCL-derived EVs on tumor progression and treatment resistance. Since EV cargo molecules play an important role in pathogenesis in DLBCL, the future research could benefit from high-throughput proteomics, transcriptomics, lipidomics, and single-vesicle analysis techniques to comprehensively characterize EV cargo. In addition, CRISPR-Cas9 technology can be used to study the functional impact of specific EV-associated genes. For experimental models, 3D tumor spheroids and patient-derived organoids may provide physiologically relevant environments while xenograft models with labeled EVs could aid in understanding their in vivo dynamics. These approaches could significantly advance the field and refine therapeutic strategies. In addition, it is important to investigate the interactions between DLBCL-derived EVs and the tumor microenvironment, especially with regard to tumor-associated macrophages, and their contributions to disease pathogenesis and treatment outcomes. Endothelial microparticles (EMPs), as a subset of EVs, are known to play significant roles in vascular homeostasis, inflammation, and coagulation in various malignancies. Their potential contributions to the tumor microenvironment, immune modulation, and angiogenesis in cancers suggest they could also influence DLBCL pathophysiology. Although previous evidence has shown that EMPs play a role in Myeloproliferative neoplasms [59], specific studies on EMPs in DLBCL are currently limited. Given these established roles in other malignancies, future research investigating the specific contributions of EMPs to DLBCL is warranted. Despite these promising findings, significant challenges hinder progress in EV research. The field is limited by variability in EV isolation methods, inconsistencies in EV purity, and sensitivity and specificity issues in cargo detection [60]. These methodological disparities contribute to discrepancies among study results, underscoring the urgent need for consensus and standardization. Considering the limitation of existing methods for tracking and imaging EVs inside cells and tissues, the creation of reliable EV tracers would improve the knowledge of intracellular and extracellular trafficking, which is essential for understanding mechanisms. The adoption of standardized protocols, such as those proposed by the International Society for Extracellular Vesicles (ISEV), is essential to enhance reproducibility and comparability across studies. Moreover, the development of novel therapeutic strategies targeting DLBCL-derived EVs could be a promising approach for the improvement of treatment outcomes for DLBCL patients, and preclinical and clinical studies are warranted to investigate the safety and efficacy of such approaches. Overall, continued research into the role of EVs in DLBCL is likely to provide valuable insights into the biology of the disease and may uncover novel therapeutic targets and strategies to improve the outcomes of DLBCL patients.

## Figures and Tables

**Table 1:** Biological functions of DLBCL-cell-derived EVs: reports from in vitro studies.

Cell Line	Method	EVs Molecule Expression	Major Findings						Interpretation	Ref.
			Proliferation	Viability	Apoptosis	Invasion	Chemoresistance	Signaling and Other Findings		
SUDHL-4	RT-qPCR	↑ miR-125b-5p							Increased miR-125b-5p in SUDHL-4-derived EVs resulted in decreasing TNFAIP3 levels, which led to decreased CD20 expression and decreased sensitivity to rituximab.	[11]
	EVs Incubated with LY8/DUL cells							↑ miR-125b-5p ↓ TNFAIP3		
	EVs treated LY-8/DUL cells and were incubated with rituximab				↓		↑	↓ CD20		
	Researchers incubated LY8/DUL cells with EVs from miR-125b-5p inhibitor transfected SUD cells and treated them with rituximab	↓ miR-125b-5p		↓				↓ miR-125b-5p ↑ TNFAIP3 ↑ CD20		
	EVs were incubated with transfected pcDNA-TNFAIP3 LY8/DUL cells followed by treatment with rituximab			↓	↑			↑ CD20		
SU-DHL-2/R (Chemoresistant) vs. SU-DHL-2	Western blot	↑ CA1							CA1 increased chemoresistance via promotion of the NF-kB and STAT3 signaling pathways.	[12]
	CA1-siRNA transfected SU-DHL-2/R treated with R-CHOP	↓ CA1					↓	↓ p-NF-kB/p65 ↓ p-STAT3/Tyr705		
OCI-LY3, OCI-LY8	qRT-PCR	↑14-3-3η							Decreased miR-107 expression in DLBCL cells led to increased 14-3-3η expression, which was also upregulated in the EVs, resulting in tumor progression.	[13]
	miR-107 agomir transfected OCI-LY3 cells		↓		↑	↓		↓ 14-3-3η		
	miR-107 antagomir transfected OCI-LY8 cells		↑		↓	↑		↑ 14-3-3η		
	Treatment with Dox									
	- OCI-LY8 cells				↑					
	- miR-107 antagomir transfected OCI-LY8 cells				↓					
OCI-LY1, SU-DHL-2	Cultured with CM of EV-incubated Mφ	NA	↑			↑ (also migration)		↑ OXPHOS in Mφ	OCI-LY1 and SU-DHL-2 cell-derived EVs enhanced OXPHOS of Mφ, thus promoting proliferation, migration, and invasion of DLBCL cells.	[14]
OCI-LY1	Western Blot								NSE secreted by DLBCL-derived EVs increased proliferation and viability and decreased apoptotic rates of lymphoma cells when co-cultured with macrophages.	[15]
	- NSE-WT (NSE-overexpressing)	↑ NSE								
	- NSE-shRNA (NSE-knockdown)	↓ NSE								
	NSE-WT LY1/DUL cells co-cultured with THP-1		↑	↑	↓ ↓ BAD, BAX ↑ BCL-2					
	NSE-WT/NSE-shRNA LY1/DUL cells		↔	↔	↔					
SU-DHL-6 SW480	Western Blot	CD20^+^ CD20^−^							CD20^+^ EVs could rescue lymphoma cells damage from rituximab.	[22]
	Treated with rituximab in addition to EVs from									
	- SU-DHL-6 cells (CD20^+^)		↑							
	- SW480 cells (CD20^−^)		↔							
OCI-LY1, Su-DHL-4, Balm3	Western blot, flow cytometry	CD20							Rituximab-induced exosome release led to the absorption of rituximab and consumption of complement on lymphoma-derived EVs.	[17]
	Treated with rituximab in the presence of complement with									
	- addition of EVs		↑				↑	↑ exosome release ↑ fixation of TCC of the exosome		
	- EV inhibitors (rapamycin/indomethacin/U18666A)		↑							
	Lentiviral sh-RNA knockdown of ABCA3	↓ ABCA3						↓ exosome release		
SU-DHL-4, OCI-LY1, OCI-LY3	Treated with Dox	↑ Dox conc in EVs						↑ exosome release	Increased ABCA3 expression was associated with an increase in exosome release, which led to reduced Dox retention and increased cell viability. Indomethacin could inhibit ABCA3 and improve drug retention.	[16]
	Treated with Dox and									
	- indomethacin	↓ ABCA3		↓			↓	↓ exosome release ↑ Dox retention in DLBCL cells		
	- Lentiviral sh-RNA knockdown of ABCA3	↓ ABCA3		↓			↓			
	Treated with pixantrone and indomethacin	↓ ABCA3		↓			↓	↑ pixantrone retention in DLBCL cells		
L540	Coincubation of CD30^+^ L540 EVs and fluorescent anti-CD30 antibody with								CD30^−^ DLBCL cell could uptake BV antibody in the presence of CD30^+^ EVs that contribute to the toxicity of BV in CD30^−^ negative cells and CD30^+^ EVs also enhanced the toxicity of BV, even in CD30^+^ cells.	[25]
	- Karpas 422 (CD30^−^)							CD30^+^ EVs internalization		
	- DoHH-2 (CD30^−^)							CD30^+^ EVs internalization		
	Incubation of BV and Karpas 422 cells (CD30^−^) without EVs			↔						
	Incubation of BV and Karpas 422 cells (CD30^−^) with addition of CD30^+^ EVs			↓						
	Incubation of BV and P30-OH/KUBO (CD30^+^) with addition of CD30^+^ EVs			↓						

Abbreviations: ABCA3, ATP-transporter A3; BAD, Bcl2-associated death promoter; BAX, Bcl2-associated X protein; Bcl2, B-cell lymphoma 2; BV, brentuximab vedotin; CA1, carbonic anhydrase 1; CM, conditional medium; Conc, concentration; DLBCL, diffuse large B-cell lymphoma; Dox, doxorubicin; EVs, extracellular vesicles; Mφ, macrophages; NA, not applicable; NSE, neuron-specific enolase; OXPHOS, oxidative phosphorylation; STAT3, signal transducer and activator of transcription 3; TCC, terminal complement complex; TNFAIP3, tumor-necrosis-factor-alpha-induced protein 3. ↑: increase or higher; ↓: decrease or lower; ↔: no change or equal.

**Table 2:** Effects of DLBCL-cell-derived EVs on macrophages.

Cell Line	EVs Molecule Expression	Method	Major Findings of Macrophages			Interpretation	Ref.
			Migration	Apoptosis	Molecule Expression and Signaling		
OCI-LY1, SU-DHL-2	NA	Mφ were treated with EVs			↑ CD163, CD206 ↑ IL-10, CCL-18 Secretion ↑ PGC-1β ↑ OXPHOS ↔ Glycolysis	EVs regulated the transformation of the M2 Mφ by increasing the expression of PGC-1β, which affected Mφ metabolism.	[14]
		shRNAs knocked down PGC-1β Mφ treated with EVs			↓ CD206 ↓ OXPHOS		
OCI-LY1	↑ NSE	THP-1 was treated with:				NSE promoted nuclear p50 translocation, downregulated IKK- β expression, and enhanced the capability of migration and differentiation toward M2 Mφ.	[15]
		- OCI-LY1 EVs			↑ p50 DNA binding activity ↓ IKK-β		
		- NSE-shRNA OCI-LY1 EVs			↓ p50 DNA binding activity		
		THP-1 was co-cultured with OCI-LY1/SU-DHL-2	↑		↑ Arg-1, IL1, IL-10, CD206 mRNA ↑ nuclear p50		
		THP-1 was transfected with p50 siRNA and co-cultured with OCI-LY1			↓ Arg-1, IL1, IL-10, CD206 mRNA		
A20	↓ miR-7e-5p	Mφ was treated with EVs from the following:				Reduced exosomal miR-7e-5p levels promoted apoptosis by targeting FasL and activated the activity of caspase cascade in M1 Mφ.	[40]
		- A20 cells with mouse M1 Mφ;		↑	↓ miR-7e-5p ↑ FasL		
		- A20 cells transfected with miR-7e-5p-mimics;		↓	↑ miR-7e-5p ↓ FasL, cleaved PARP, caspase3		
		- A20 cells transfected with miR-7e-5p-mimics with GW4869 added (to inhibit EV secretion)			↑ FasL, cleaved PARP, caspase3		

Abbreviations: DLBCL, diffuse large B-cell lymphoma; EVs, extracellular vesicles; Mφ, macrophages; NA, not applicable; NSE, neuron-specific enolase; OXPHOS, oxidative phosphorylation; PARP, poly (ADP-ribose) polymerase. ↑: increase or higher; ↓: decrease or lower; ↔: no change or equal.

**Table 3:** Role of DLBCL-cell-derived Evs: report from in vivo studies.

Model	Xenograft Cell Line	Evs Cell	Method	Molecule Expression	Major Findings					Interpretation	Ref.
					Tumor Volume	Apoptosis	Migration	Angiogenesis	Microenvironment and Immunohistology		
NOD-SCID mice (6 weeks)	OCI-LY3	OCI-LY3	Coinjection of OCI-LY3 with Evs		↑		↑	↑		DLBCL-derived Evs promoted tumor growth and enhanced migration and angiogenesis.	[42]
Nude mice (5–6 weeks)	OCI-LY8	SUDHL-4	Mice were injected with Evs + 10 mg/kg rituximab	↑ miR-125b-5p	↑				↓ TNFAIP3 ↓ CD20	Evs reduced the sensitivity of DLBCL to rituximab via miR-125b-5p, which regulated TNFAIP3, thereby affecting CD20 expression.	[11]
SCID beige mice (3–4 weeks)	OCI-LY8	-	OCI-LY8 were transfected with miR-107 Agomir NC	↓ miR-107	↑					Downregulated miR-107 expression increased tumor growth.	[13]
BALB/c nude mice (4 weeks)	SU-DHL-2	-	Mice were pretreated with Evs from the following: - CA1-siRNA-transfected SU-DHL-2/R	↓ CA1	↓				↓ NF-kB ↓ STAT3	Exosomal CA1 reduced chemotherapy sensitivity via NF-kB and STAT3 signaling pathways.	[12]
BALB/c nu/nu mice (6 weeks)	OCI-LY1	-	NSE-WT	↑ NSE	↑				↑ CD68^+^ CD206^+^ M2 Mφ ↑ Ki67	NSE overexpression increased tumor size and several CD68^+^ CD206^+^ M2 Mφ levels	[15]
			NSE-shRNA1	↓ NSE	↓	↑			↓ Ki67		
CAM	OCI-Ly1, SU-DHL-4	-	Pretreatment with the following:1 mL of 10 µmol/L indomethacin: - 50 µg Dox or pixantrone		↓				↑ Distorted architecture ↓ Tumor cell density ↑ Dox retention in tumor cell nuclei	Indomethacin pretreatment was associated with drug retention and shifting of drugs from cytoplasm to nucleus and resulted in increased efficacy of doxorubicin and pixantrone.	[16]
BALB/C mice (aged 6 weeks)	A20	A20	Immunization with A20 EV-pulsed DCC: - IV/DCC^Evs^ 10 µg/mouse weekly/three doses	DCC^Evs^ (exosome-based tumor vaccine)					↑ CD8^+^ T cells ↓ CD4^+^ CD25^+^ FOXP3^+^ Treg cells ↑ cytolysis efficiency in the spleen and lymph node lymphocytes	The activated DCC^Evs^ triggered an anti-tumor immune response and induced anti-tumor activity.	[42]

Abbreviations: CA1, carbonic anhydrase 1; CAM, chorioallantoic membrane; DCC, dendritic cells; Dox, doxorubicin; EVs, extracellular vesicles; FOXP3, forkhead box P3; Mφ, macrophage; SCID, severe combined immunodeficiency; NSE, neuron-specific enolase; STAT3: signal transducer and activator of transcription 3; TNFAIP3, tumor-necrosis-factor-alpha-induced protein 3. ↑: increase or higher; ↓: decrease or lower.

**Table 4:** Association between circulating EVs and clinicopathologic features in DLBCL patients.

Subjects (N)	Source of EVs	EV Extraction Method	Detection Method	Biomarker Expression	Clinicopathologic Features						Interpretation	Ref.
					COO	Ki67PI	Clinical	Stage	IPI	Laboratory		
DLBCL (164)	Plasma	exoRNeasy Plasma Midi Kit	SiMoa with CD9-CD63 assays	↑ CD9-CD63	GCB		≥2 extranodal sites				High CD9-63 signals associated with GCB subtype and ≥2 extranodal sites.	[43]
			SiMoa with PD-L1-CD63 assays	↑ PD-L1-CD63			≥2 extranodal sites				High PD-L1-CD63 signals associated with ≥2 extranodal sites.	
DLBCL (42)	Plasma	exoEasy Maxi kit	qRT-PCR	↓ miR-107			↑ ECOG score	↑	↑	↑ LDH ↑ B2M	Decreased exosomal miR-107 showed poor prognostic relevance.	[13]
DLBCL (112)	Serum	ExoQuick-TC kit	Western blot	↑ CA1					↑		The expression level of CA1 was higher in exosomes from chemoresistant patients and correlated with higher IPI scores.	[12]
DLBCL (48)	Plasma	ExoSpin Exosome Purification Kit	qRT-PCR	↔ miR-146a-5p					↔	↔ LDH	No correlation of miR-146a-5p with LDH and IPI score.	[44]
DLBCL (48)	Plasma	ExoSpin Exosome Purification Kit	qRT-PCR	↔ miR-155		↔	↔ age, gender	↔	↔	↔ LDH	No correlation between miR-155, Let-7g, and Let-7i with age, gender, LDH, IPI, and disease stage.	[45]
				↔ Let-7g		↔	↔ age, gender	↔	↔	↔ LDH		
				↔ Let-7i		↔	↔ age, gender	↔	↔	↔ LDH		
DLBCL (116)	Serum	ExoQuick Kit	qRT-PCR	↔ miR-99a-5p	↔		↔ age, gender ↔ B symptoms	↔	↔	↔ LDH	No correlation between miR-99a-5p and miR-125b-5p with COO, age, gender, B symptoms, LDH, IPI score, and disease stage.	[46]
				↔ miR-125b-5p	↔		↔ age, gender ↔ B symptoms	↔	↔	↔ LDH		

Abbreviations: CA1, carbonic anhydrase 1; DLBCL, diffuse large B-cell lymphoma; EVs, extracellular vesicles; GCB, germinal-center B-cell; IPI, International Prognostic Index; Ki67PI, Ki67 proliferation index; LDH, lactate dehydrogenase; SiMoa, single molecule array. ↑: increase or higher; ↓: decrease or lower; ↔: no change or equal.

**Table 5:** The use of EVs as potential diagnostic markers.

Populations (N)	Source of EVs	EV Extraction Method	Detection Method	Index Test	Diagnostic Indices		Interpretation	Ref.
					Molecule Expression	AUC		
DLBCL (164) Controls (25)	Plasma	exoRNeasy Plasma Midi Kit	SiMoa with CD9-CD63 assays	CD9-CD63	↑	0.99	Exosomal CD9-CD63 and PD-L1-CD63 in DLBCL patients were significantly higher and able to differentiate DLBCL patients from healthy controls.	[43]
			SiMoa with PD-L1-CD63 assays	PD-L1-CD63	↑	0.87		
DLBCL (42) Controls (31)	Plasma	exoEasy Maxi kit	qRT-PCR	miR-107	↓	0.854	Plasma exosomal miR-107, miR-375-3p, and miR-485-3p could be used to distinguish DLBCL patients from normal controls.	[13]
				miR-375-3p	↓	0.769		
				miR-485-3p	↑	0.739		
				14-3-3η	↑	-	Expression of 14-3-3η was upregulated in plasma exosomes of DLBCL patients.	
DLBCL (89) Control (48)	Serum	ExoQuick Kit	qRT-PCR	miR-451a	↓	0.7147	Exosomal miR-451a in DLBCL patients was significantly lower and could be used to distinguish DLBCL patients from normal controls.	[53]
DLBCL (48) Control (6)	Plasma	ExoSpin Exosome Purification Kit	qRT-PCR	miR-146a-5p	↔	-	No significant difference in the level of expression of miR-146a-5p compared to controls was noted.	[44]
DLBCL (5) Controls (18)	Serum	DC	qRT-PCR	miR-155	↔	-	No difference in the level of expression of miR-155 compared to controls.	[54]

Abbreviations: DC, differential centrifugation; DLBCL, diffuse large B-cell lymphoma; EVs, extracellular vesicles; SiMoa, single molecule array. ↑: increase or higher; ↓: decrease or lower; ↔: no change or equal.

**Table 6:** Prognostic prediction value and treatment efficacy of EVs.

Subject (N)	Source of EVs	EV Extraction Method	Detection Method	Index Test	Expression Level	Aim of Test	Index				Interpretation	Ref.
							AUC	PFS	OS	CR Rate		
DLBCL (42)	Plasma	exoEasy Maxi kit	qRT-PCR	miR-107	↓	Predicted PFS	-	↓	-	-	Low expression of exosomal miR-107 correlated with poor PFS.	[13]
DLLBCL (164)	Plasma	exoRNeasy Plasma Midi Kit	SiMoa with CD9-CD63/ PD-L1-CD63 assays	CD9-CD63/ PD-L1-CD63	↑	Predicted prognosis and treatment response	-	↓ 5-year	↓ 5-year	↓	Elevated CD9-CD63 and PD-L1-CD63 signals were associated with poor PFS, OS, and treatment response.	[43]
DLBCL - R (31) - S (81)	Serum	ExoQuick-TC kit	Western Blot	CA1	↑	Predicted CMT efficacy	0.812	↓	-	-	Higher expression of CA1 in chemoresistant patients and combination of exosomal CA1 with IPI score provide slightly superior prediction performance than exosomal CA1 alone.	[12]
				CA1 plus IPI score	↑		0.821	-	-	-		
DLBCL (116) - R (33) - S (83)	Serum	ExoQuick Kit	qRT-PCR	miR-99a-5p	↑	Predicted PFS and treatment response	0.744	↓	-	↓	High expression of exosomal miR-99a-5p and miR-125b-5p had high accuracy in predicting chemoresistance of DLBCL patients, contributed to poor PFS, and, combined with IPI, can give higher accuracy.	[46]
				miR-125b-5p	↑		0.780	↓	-	↓		
				miR-99a-5p and IPI score			0.833	-	-	-		
				miR-125b-5p and IPI score			0.814	-	-	-		
DLBCL **- **S (73) - R (25)	Serum	ExoQuick Kit	qRT-PCR	miR-451a	↑ after treatment	Changes in serum exosome miR-451a during treatment and therapeutic effects	0.8038	-	-	-	Exosomal miR-451a could act as an indicator for evaluating treatment efficacy.	[53]
DLBCL - R/R (16) - Responsive (17) - Receiving R-CHOP (15)	Plasma	ExoSpin	qRT-PCR	miR-155	↑ R/R > responsive > receiving	Changes in plasma exosome miR-155 and Let-7g in different groups of patients	-	-	-	-	R/R patients had significantly increased exosomal miR-155 levels and this miRNA may be useful as a prognostic marker to predict response treatment. Decreased level of exosomal Let-7g might be useful as a predictor of response to treatment.	[45]
DLBCL - Relapsed (8) - Responsive (17) - Receiving R- CHOP (15)	Plasma	ExoSpin	qRT-PCR	miR-155	↑ Relapsed > responsive > receiving		-	-	-	-		
DLBCL - Refractory (8) - Responsive (17) - Receiving R-CHOP (15)				miR-155	↑ Refractory > responsive > receiving		-	-	-	-		
DLBCL - R/R (16) - Responsive (17) - Receiving R-CHOP (15)				Let-7g	↓ Receiving < responsive < R/R		-	-	-	-		
DLBCL - Receiving R-CHOP (15) - R/R (16) - Responsive (17)				Let-7i	↔		-	-	-	-		
DLBCL (48) - Refractory (16) - Response (17)	Plasma	ExoSpin Exosome Purification Kit	qRT-PCR	miR-146a-5p	↔	Differentiated between responsive and refractory patients	-	-	-	-	No significant difference in the level of expression of exosomal miR-146a between refractory patients compared to responsive patients was noted.	[44]

Abbreviations: AUC, area under the curve, CA1, carbonic anhydrase 1; CR, complete remission; DLBCL, diffuse large B-cell lymphoma; IPI, International Prognostic Index; OS, overall survival; PFS, progression-free survival; R, chemoresistant; R/R, relapsed refractory; S, chemo-sensitive; SiMoa, single molecule array. ↑: increase or higher; ↓: decrease or lower; ↔: no change or equal.

## References

[1] Sehn L.H., Salles G. (2021). Diffuse Large B-Cell Lymphoma. N. Engl. J. Med..

[2] Coiffier B., Thieblemont C., Van Den Neste E., Lepeu G., Plantier I., Castaigne S., Lefort S., Marit G., Macro M., Sebban C. (2010). Long-term outcome of patients in the LNH-98.5 trial, the first randomized study comparing rituximab-CHOP to standard CHOP chemotherapy in DLBCL patients: A study by the Groupe d’Etudes des Lymphomes de l’Adulte. Blood.

[3] Pfreundschuh M., Trümper L., Osterborg A., Pettengell R., Trneny M., Imrie K., Ma D., Gill D., Walewski J., Zinzani P.-L. (2006). CHOP-like chemotherapy plus rituximab versus CHOP-like chemotherapy alone in young patients with good-prognosis diffuse large-B-cell lymphoma: A randomised controlled trial by the MabThera International Trial (MInT) Group. Lancet Oncol..

[4] Pfreundschuh M., Schubert J., Ziepert M., Schmits R., Mohren M., Lengfelder E., Reiser M., Nickenig C., Clemens M., Peter N. (2008). Six versus eight cycles of bi-weekly CHOP-14 with or without rituximab in elderly patients with aggressive CD20+ B-cell lymphomas: A randomised controlled trial (RICOVER-60). Lancet Oncol..

[5] Théry C., Witwer K.W., Aikawa E., Alcaraz M.J., Anderson J.D., Andriantsitohaina R., Antoniou A., Arab T., Archer F., Atkin-Smith G.K. (2018). Minimal information for studies of extracellular vesicles 2018 (MISEV2018): A position statement of the International Society for Extracellular Vesicles and update of the MISEV2014 guidelines. J. Extracell. Vesicles.

[6] Lötvall J., Hill A.F., Hochberg F., Buzás E.I., Di Vizio D., Gardiner C., Gho Y.S., Kurochkin I.V., Mathivanan S., Quesenberry P. (2014). Minimal experimental requirements for definition of extracellular vesicles and their functions: A position statement from the International Society for Extracellular Vesicles. J. Extracell. Vesicles.

[7] Yáñez-Mó M., Siljander P.R., Andreu Z., Zavec A.B., Borràs F.E., Buzas E.I., Buzas K., Casal E., Cappello F., Carvalho J. (2015). Biological properties of extracellular vesicles and their physiological functions. J. Extracell. Vesicles.

[8] Zaborowski M.P., Balaj L., Breakefield X.O., Lai C.P. (2015). Extracellular Vesicles: Composition, Biological Relevance, and Methods of Study. Bioscience.

[9] Zhang X., Liu D., Gao Y., Lin C., An Q., Feng Y., Liu Y., Liu D., Luo H., Wang D. (2021). The Biology and Function of Extracellular Vesicles in Cancer Development. Front. Cell Dev. Biol..

[10] Chang W.H., Cerione R.A., Antonyak M.A. (2021). Extracellular Vesicles and Their Roles in Cancer Progression. Methods Mol. Biol..

[11] Zhang L., Zhou S., Zhou T., Li X., Tang J. (2021). Potential of the tumor-derived extracellular vesicles carrying the miR-125b-5p target TNFAIP3 in reducing the sensitivity of diffuse large B cell lymphoma to rituximab. Int. J. Oncol..

[12] Feng Y., Zhong M., Tang Y., Liu X., Liu Y., Wang L., Zhou H. (2020). The Role and Underlying Mechanism of Exosomal CA1 in Chemotherapy Resistance in Diffuse Large B Cell Lymphoma. Mol. Ther. Nucleic Acids.

[13] Liu J., Han Y., Hu S., Cai Y., Yang J., Ren S., Zhao Y., Lu T., Zhou X., Wang X. (2021). Circulating Exosomal MiR-107 Restrains Tumorigenesis in Diffuse Large B-Cell Lymphoma by Targeting 14-3-3η. Front. Cell Dev. Biol..

[14] Liu W., Zhu M., Wang H., Wang W., Lu Y. (2020). Diffuse large B cell lymphoma-derived extracellular vesicles educate macrophages to promote tumours progression by increasing PGC-1β. Scand. J. Immunol..

[15] Zhu M.Y., Liu W.J., Wang H., Wang W.D., Liu N.W., Lu Y. (2019). NSE from diffuse large B-cell lymphoma cells regulates macrophage polarization. Cancer Manag. Res..

[16] Koch R., Aung T., Vogel D., Chapuy B., Wenzel D., Becker S., Sinzig U., Venkataramani V., von Mach T., Jacob R. (2016). Nuclear Trapping through Inhibition of Exosomal Export by Indomethacin Increases Cytostatic Efficacy of Doxorubicin and Pixantrone. Clin. Cancer Res..

[17] Aung T., Chapuy B., Vogel D., Wenzel D., Oppermann M., Lahmann M., Weinhage T., Menck K., Hupfeld T., Koch R. (2011). Exosomal evasion of humoral immunotherapy in aggressive B-cell lymphoma modulated by ATP-binding cassette transporter A3. Proc. Natl. Acad. Sci. USA.

[18] Frenquelli M., Tonon G. (2020). WNT Signaling in Hematological Malignancies. Front. Oncol..

[19] Ge X., Lv X., Feng L., Liu X., Wang X. (2012). High expression and nuclear localization of β-catenin in diffuse large B-cell lymphoma. Mol. Med. Rep..

[20] Koch R., Demant M., Aung T., Diering N., Cicholas A., Chapuy B., Wenzel D., Lahmann M., Güntsch A., Kiecke C. (2014). Populational equilibrium through exosome-mediated Wnt signaling in tumor progression of diffuse large B-cell lymphoma. Blood.

[21] Li J., Gao N., Gao Z., Liu W., Pang B., Dong X., Li Y., Fan T. (2021). The Emerging Role of Exosomes in Cancer Chemoresistance. Front. Cell Dev. Biol..

[22] Oksvold M.P., Kullmann A., Forfang L., Kierulf B., Li M., Brech A., Vlassov A.V., Smeland E.B., Neurauter A., Pedersen K.W. (2014). Expression of B-cell surface antigens in subpopulations of exosomes released from B-cell lymphoma cells. Clin. Ther..

[23] Nagel D., Vincendeau M., Eitelhuber A.C., Krappmann D. (2014). Mechanisms and consequences of constitutive NF-κB activation in B-cell lymphoid malignancies. Oncogene.

[24] Pasqualucci L., Zhang B. (2016). Genetic drivers of NF-κB deregulation in diffuse large B-cell lymphoma. Semin. Cancer Biol..

[25] Lobastova L., Lettau M., Babatz F., de Oliveira T.D., Nguyen P.H., Pauletti B.A., Schauss A.C., Dürkop H., Janssen O., Leme A.F.P. (2021). CD30-Positive Extracellular Vesicles Enable the Targeting of CD30-Negative DLBCL Cells by the CD30 Antibody-Drug Conjugate Brentuximab Vedotin. Front. Cell Dev. Biol..

[26] Ribeiro Franco P.I., Rodrigues A.P., de Menezes L.B., Pacheco Miguel M. (2020). Tumor microenvironment components: Allies of cancer progression. Pathol. Res. Pract..

[27] Kridel R., Steidl C., Gascoyne R.D. (2015). Tumor-associated macrophages in diffuse large B-cell lymphoma. Haematologica.

[28] Scott D.W., Gascoyne R.D. (2014). The tumour microenvironment in B cell lymphomas. Nat. Rev. Cancer.

[29] Cioroianu A.I., Stinga P.I., Sticlaru L., Cioplea M.D., Nichita L., Popp C., Staniceanu F. (2019). Tumor Microenvironment in Diffuse Large B-Cell Lymphoma: Role and Prognosis. Anal. Cell. Pathol..

[30] Li Y.-L., Shi Z.-H., Wang X., Gu K.-S., Zhai Z.-M. (2019). Tumor-associated macrophages predict prognosis in diffuse large B-cell lymphoma and correlation with peripheral absolute monocyte count. BMC Cancer.

[31] Cai Q.C., Liao H., Lin S.X., Xia Y., Wang X.X., Gao Y., Lin Z.-X., Lu J.-B., Huang H.-Q. (2012). High expression of tumor-infiltrating macrophages correlates with poor prognosis in patients with diffuse large B-cell lymphoma. Med. Oncol..

[32] Wada N., Zaki M.A., Hori Y., Hashimoto K., Tsukaguchi M., Tatsumi Y., Ishikawa J., Tominaga N., Sakoda H., Take H. (2012). Tumour-associated macrophages in diffuse large B-cell lymphoma: A study of the Osaka Lymphoma Study Group. Histopathology.

[33] Nam S.J., Go H., Paik J.H., Kim T.M., Heo D.S., Kim C.W., Jeon Y.C. (2014). An increase of M2 macrophages predicts poor prognosis in patients with diffuse large B-cell lymphoma treated with rituximab, cyclophosphamide, doxorubicin, vincristine and prednisone. Leuk. Lymphoma.

[34] Marchesi F., Cirillo M., Bianchi A., Gately M., Olimpieri O.M., Cerchiara E., Renzi D., Micera A., Balzamino B.O., Bonini S. (2015). High density of CD68+/CD163+ tumour-associated macrophages (M2-TAM) at diagnosis is significantly correlated to unfavorable prognostic factors and to poor clinical outcomes in patients with diffuse large B-cell lymphoma. Hematol. Oncol..

[35] Pan Y., Yu Y., Wang X., Zhang T. (2020). Tumor-Associated Macrophages in Tumor Immunity. Front. Immunol..

[36] Chávez-Galán L., Olleros M.L., Vesin D., Garcia I. (2015). Much More than M1 and M2 Macrophages, There are also CD169+ and TCR+ Macrophages. Front. Immunol..

[37] Wang L., Liu P., Chen X., Geng Q., Lu Y. (2012). Serum neuron-specific enolase is correlated with clinical outcome of patients with non-germinal center B cell-like subtype of diffuse large B-cell lymphoma treated with rituximab-based immunochemotherapy. Med. Oncol..

[38] Wang L., Liu P., Geng Q., Chen X., Lv Y. (2011). Prognostic significance of neuron-specific enolase in patients with diffuse large B-cell lymphoma treated with rituximab-based immunochemotherapy. Leuk. Lymphoma.

[39] Mizuno K., Ogura S., Kamiya T., Ito C., Fujita Y., Aisa Y., Nakazato T. (2018). Prognostic Significance of Neuron-Specific Enolase in Patients with Malignant Lymphoma. Blood.

[40] Lou X., Fu J., Zhao X., Zhuansun X., Rong C., Sun M., Niu H., Wu L., Zhang Y., An L. (2020). MiR-7e-5p downregulation promotes transformation of low-grade follicular lymphoma to aggressive lymphoma by modulating an immunosuppressive stroma through the upregulation of FasL in M1 macrophages. J. Exp. Clin. Cancer Res..

[41] Xiang X., Wang J., Lu D., Xu X. (2021). Targeting tumor-associated macrophages to synergize tumor immunotherapy. Signal Transduct. Target. Ther..

[42] Chen Z., You L., Wang L., Huang X., Liu H., Wei J.Y., Zhu L., Qian W. (2018). Dual effect of DLBCL-derived EXOs in lymphoma to improve DC vaccine efficacy in vitro while favor tumorgenesis in vivo. J. Exp. Clin. Cancer Res..

[43] Li J.W., Shi D., Wan X.C., Hu J., Su Y.F., Zeng Y.P., Hu Z.-J., Yu B.-H., Zhang Q.-L., Wei P. (2021). Universal extracellular vesicles and PD-L1+ extracellular vesicles detected by single molecule array technology as circulating biomarkers for diffuse large B cell lymphoma. Oncoimmunology.

[44] Zare N., Eskandari N., Mehrzad V., Javanmard S.H. (2019). The expression level of hsa-miR-146a-5p in plasma-derived exosomes of patients with diffuse large B-cell lymphoma. J. Res. Med. Sci..

[45] Zare N., Haghjooy Javanmard S., Mehrzad V., Eskandari N., Kefayat A. (2019). Evaluation of exosomal miR-155, let-7g and let-7i levels as a potential noninvasive biomarker among refractory/relapsed patients, responsive patients and patients receiving R-CHOP. Leuk. Lymphoma.

[46] Feng Y., Zhong M., Zeng S., Wang L., Liu P., Xiao X., Liu Y. (2019). Exosome-derived miRNAs as predictive biomarkers for diffuse large B-cell lymphoma chemotherapy resistance. Epigenomics.

[47] Sun C., Jia Y., Wang W., Bi R., Wu L., Bai Q., Zhou X. (2019). Integrative analysis of PD-L1 DNA status, mRNA status and protein status, and their clinicopathological correlation, in diffuse large B-cell lymphoma. Histopathology.

[48] Kataoka K., Miyoshi H., Sakata S., Dobashi A., Couronné L., Kogure Y., Sato Y., Nishida K., Gion Y., Shiraishi Y. (2019). Frequent structural variations involving programmed death ligands in Epstein-Barr virus-associated lymphomas. Leukemia.

[49] De Mattos-Arruda L., Siravegna G. (2021). How to use liquid biopsies to treat patients with cancer. ESMO Open.

[50] Jin Y., Chen K., Wang Z., Wang Y., Liu J., Lin L., Shao Y., Gao L., Yin H., Cui C. (2016). DNA in serum extracellular vesicles is stable under different storage conditions. BMC Cancer.

[51] Matthiesen R., Gameiro P., Henriques A., Bodo C., Moraes M.C.S., Costa-Silva B., Cabeçadas J., da Silva M.G., Beck H.C., Carvalho A.S. (2022). Extracellular Vesicles in Diffuse Large B Cell Lymphoma: Characterization and Diagnostic Potential. Int. J. Mol. Sci..

[52] Rutherford S.C., Fachel A.A., Li S., Sawh S., Muley A., Ishii J., Saxena A., Dominguez P.M., Lopes E.C., Agirre X. (2018). Extracellular vesicles in DLBCL provide abundant clues to aberrant transcriptional programming and genomic alterations. Blood.

[53] Xiao X.B., Gu Y., Sun D.L., Ding L.Y., Yuan X.G., Jiang H.W., Wu Z.-X. (2019). Effect of rituximab combined with chemotherapy on the expression of serum exosome miR-451a in patients with diffuse large b-cell lymphoma. Eur. Rev. Med. Pharmacol. Sci..

[54] Caivano A., La Rocca F., Simeon V., Girasole M., Dinarelli S., Laurenzana I., De Stradis A., De Luca L., Trino S., Traficante A. (2017). MicroRNA-155 in serum-derived extracellular vesicles as a potential biomarker for hematologic malignancies-a short report. Cell. Oncol..

[55] Yazarlou F., Kadkhoda S., Ghafouri-Fard S. (2021). Emerging role of let-7 family in the pathogenesis of hematological malignancies. Biomed. Pharmacother..

[56] Trabolsi A., Arumov A., Schatz J.H. (2024). Bispecific antibodies and CAR-T cells: Dueling immunotherapies for large B-cell lymphomas. Blood Cancer J..

[57] Stawarska A., Bamburowicz-Klimkowska M., Runden-Pran E., Dusinska M., Cimpan M.R., Rios-Mondragon I., Grudzinski I.P. (2024). Extracellular Vesicles as Next-Generation Diagnostics and Advanced Therapy Medicinal Products. Int. J. Mol. Sci..

[58] Kumar M.A., Baba S.K., Sadida H.Q., Marzooqi S.A., Jerobin J., Altemani F.H., Algehainy N., Alanazi M.A., Abou-Samra A.-B., Kumar R. (2024). Extracellular vesicles as tools and targets in therapy for diseases. Signal Transduct. Target. Ther..

[59] Todor S.B., Ichim C., Boicean A., Mihaila R.G. (2024). Cardiovascular Risk in Philadelphia-Negative Myeloproliferative Neoplasms: Mechanisms and Implications-A Narrative Review. Curr. Issues Mol. Biol..

[60] De Sousa K.P., Rossi I., Abdullahi M., Ramirez M.I., Stratton D., Inal J.M. (2023). Isolation and characterization of extracellular vesicles and future directions in diagnosis and therapy. Wiley Interdiscip. Rev. Nanomed. Nanobiotechnol..

